# Prediction of mortality using a multi-bed vascular calcification score in the Diabetes Heart Study

**DOI:** 10.1186/s12933-014-0160-5

**Published:** 2014-12-12

**Authors:** Amanda J Cox, Fang-Chi Hsu, Subhashish Agarwal, Barry I Freedman, David M Herrington, J Jeffrey Carr, Donald W Bowden

**Affiliations:** Center for Diabetes Research, Wake Forest School of Medicine, Winston-Salem, NC USA; Center for Genomics and Personalized Medicine Research, Wake Forest School of Medicine, 27157 Winston-Salem, NC USA; Department of Biochemistry, Wake Forest School of Medicine, Winston-Salem, NC USA; Molecular Basis of Disease, Griffith Health Institute, Griffith University, Southport, QLD Australia; Heart Foundation Research Center, Griffith Health Institute, Griffith University, Southport, QLD Australia; School of Medical Science, Griffith University, Southport, QLD Australia; Department of Biostatistical Sciences, Wake Forest School of Medicine, Winston-Salem, NC USA; Division of Electrophysiology, Section of Cardiology, Northwestern University Feinberg School of Medicine, Chicago, IL USA; Department of Internal Medicine - Nephrology, Wake Forest School of Medicine, Winston-Salem, NC USA; Department of Internal Medicine - Cardiology, Wake Forest School of Medicine, Winston-Salem, NC USA; Department of Radiologic Sciences, Wake Forest School of Medicine, Winston-Salem, NC USA

**Keywords:** Vascular calcified plaque, Mortality, Computed tomography, Type 2 diabetes

## Abstract

**Background:**

Vascular calcified plaque, a measure of subclinical cardiovascular disease (CVD), is unlikely to be limited to a single vascular bed in patients with multiple risk factors. Consideration of vascular calcified plaque as a global phenomenon may allow for a more accurate assessment of the CVD burden. The aim of this study was to examine the utility of a combined vascular calcified plaque score in the prediction of mortality.

**Methods:**

Vascular calcified plaque scores from the coronary, carotid, and abdominal aortic vascular beds and a derived multi-bed score were examined for associations with all-cause and CVD-mortality in 699 European-American type 2 diabetes (T2D) affected individuals from the Diabetes Heart Study. The ability of calcified plaque to improve prediction beyond Framingham risk factors was assessed.

**Results:**

Over 8.4 ± 2.3 years (mean ± standard deviation) of follow-up, 156 (22.3%) participants were deceased, 74 (10.6%) from CVD causes. All calcified plaque scores were significantly associated with all-cause (HR: 1.4-1.8; p < 1x10^−5^) and CVD-mortality (HR: 1.5-1.9; p < 1×10^−4^) following adjustment for Framingham risk factors. Associations were strongest for coronary calcified plaque. Improvement in prediction of outcome beyond Framingham risk factors was greatest using coronary calcified plaque for all-cause mortality (AUC: 0.720 to 0.757, p = 0.004) and the multi-bed score for CVD mortality (AUC: 0.731 to 0.767, p = 0.008).

**Conclusions:**

Although coronary calcified plaque and the multi-bed score were the strongest predictors of all-cause mortality and CVD-mortality respectively in this T2D-affected sample, carotid and abdominal aortic calcified plaque scores also significantly improved prediction of outcome beyond traditional risk factors and should not be discounted as risk stratification tools.

**Electronic supplementary material:**

The online version of this article (doi:10.1186/s12933-014-0160-5) contains supplementary material, which is available to authorized users.

## Background

As the major cause of mortality in Western industrialized countries, cardiovascular disease (CVD) accounts for approximately 35% of all-cause mortality [1]. Compared to non-diabetic individuals, those with type 2 diabetes (T2D) experience a two-fold increased risk for coronary artery disease with up to 65% of all-cause mortality attributed to CVD [1,2]. However, the risk profile is not uniform for all T2D-affected individuals [3-6] and ongoing risk assessment is essential if individualized management strategies are to be implemented. Thus, identification of predictors for CVD events and mortality in high-risk individuals with T2D offers opportunities for improved clinical management.

Coronary artery calcified plaque (CAC), determined by computed tomography scanning, is a measure of CVD burden [7,8]. CAC scores have been shown to be an independent predictor of CVD outcomes and mortality in population-based studies [9-11] and a powerful predictor of CVD events [12,13], and all-cause and CVD-mortality in T2D-affected individuals [4,14-18]. In the family-based Diabetes Heart Study (DHS) we have previously reported that CAC scores are associated with up to 6-fold [6] and 11-fold [19] elevations in risk for all-cause mortality and CVD-mortality, respectively. However, in T2D-affected individuals where the CVD risk factor burden is likely to be high, vascular calcified plaque is unlikely to be limited to a single vascular bed and consideration of vascular calcified plaque as a global phenomenon may allow for a more comprehensive assessment of the burden of disease.

Few studies report vascular calcified plaque beyond a single vascular bed, although methods used for CAC also allow quantification of carotid artery calcified plaque (CarCP) and aortic calcified plaque [20-24]. Allison et al., recently compared utility of CAC, CarCP and AACP in prediction of mortality in a general population cohort with T2D prevalence of ~3% and surprisingly found that calcified plaque in vascular beds other than the coronary arteries better predicted outcome following adjustment for other CVD risk factors [25]. We sought to perform a similar comparison in DHS sample where T2D prevalence is approximately 85%. We were also interested to examine the utility of combined score of vascular calcified plaque from three different vascular beds (CAC, CarCP, AACP) in the prediction of outcome.

## Methods

### Study design and sample

The DHS cohort includes 1,220 self-described European American individuals from 475 families. Briefly, the DHS recruited siblings concordant for T2D without advanced renal insufficiency, manifesting as serum creatinine concentration >2 mg/dL or end-stage renal disease. When possible, one non-T2D affected sibling was also recruited. T2D was clinically defined as diabetes developing after the age of 35 years and treated initially with diet and exercise and/or oral anti-hyperglycemic medications. Diagnoses were confirmed by baseline measurement of fasting blood glucose and glycosylated hemoglobin (HbA1c). Full ascertainment and recruitment criteria have been previously described [26,27].

Study protocols were approved by the Institutional Review Board at Wake Forest School of Medicine, and all participants provided written informed consent. Participant examinations were conducted in the General Clinical Research Center of the Wake Forest Baptist Medical Center. Examinations included interview to record for medical history, health behaviors and medication use, anthropometric measurements, resting blood pressure (BP), electrocardiography and fasting blood sampling for laboratory analyses including fasting glucose, HbA_1C_, lipids, serum albumin and creatinine concentration. Individuals self-reported history of prior CVD based on prior events (angina, myocardial infarction and stroke) and/or interventions (coronary artery angioplasty/stenting/bypass grafting or carotid artery endarterectomy). Individuals were classified as (i) hypertensive if prescribed anti-hypertensive medication or if BP measurements exceeded 140 mmHg (systolic) or 90 mmHg (diastolic) and (ii) dyslipidemic based on the criteria established in the Third Report of the National Cholesterol Education Program Expert Panel Detection, Evaluation and Treatment in Adults (ATP III) [28].

### Vascular calcified plaque

Subclinical CVD was assessed by measurement of calcified plaque in the coronary (CAC), carotid (CarCP) and abdominal aortic (AACP) vascular beds with calcium scores calculated as previously described [29-32]. Briefly all computed tomography (CT) examinations were performed on a single-slice helical CT or a four-channel multidetector CT both with cardiac gating and capable of 500 ms temporal resolution (HiSpeed LX and LightSpeed QXi with the SmartScore Cardiac scan package; General Electric Medical Systems, Waukesha, WI). For CAC, after a scout image of the chest, the heart was imaged during suspended respiration at end inspiration. Scan parameters were 3 mm slice thickness, 26 cm display field of view, retrospective cardiac gating, 120 KV, 240 mA, and CT scan pitch adjusted to heart rate for the single-slice system and 2.5-mm slice thickness in four-slice mode and prospective cardiac gating at 50% of the RR interval, for the multidetector CT. For CarCP an unenhanced CT scan was performed through the neck from C2-3 to the C6-7 disc levels covering the right and left carotid bifurcations. Specifically, the carotid bifurcation level was identified, and the 15 mm of internal and external carotid above the bifurcation and 30 mm of carotid bulb and common carotid artery below the bifurcation were measured. Scan parameters were 3 mm slice thickness, 18 cm field of view, 120 KV and 280 mA. For the AACP, an un-enhanced scan of the abdomen was performed. For AACP, the entire juxtarenal and infrarenal aorta, starting 25 mm proximal to the origin of the superior mesenteric artery and extending 25mm into the common iliac arteries below the aortic bifurcation, was measured. Scan parameters were 2.5 mm slice thickness, 35 cm field of view, 120 kV, 250 mA.

To assess vascular calcified plaque from a more global perspective, a multi-bed vascular calcification score was derived (multi-bed score) using an approach similar to that described previously for assessment of adiposity from multiple sites [33]. First available calcified plaque scores from each of the three vascular beds (coronary, carotid and abdominal aorta) were obtained. To account for the differences in the absolute values between the three vascular beds (by virtue of the natural differences in the sizes of the vessels - coronary artery versus carotid artery versus abdominal aorta), the distributions of each were then standardized. Standardized scores were calculated by determining the difference between overall mean for that vascular bed and each individual calcified plaque score and dividing this value by the standard deviation. The distribution of each of the standardized CAC, CarCP and AACP scores was then fixed to minimum value of zero producing a set of positive values for each of the three beds. These values were summed for each individual to determine the multi-bed score.

### Vital status

Vital status was determined for all participants from the National Social Security Death Index maintained by the United States Social Security Administration. For participants confirmed as deceased, length of follow-up was determined from the date of initial study visit to date of death. For all other participants, the length of follow-up was determined from the date of the initial study visit to the end of 2012. For deceased participants, copies of death certificates were obtained from relevant county Vital Records Offices to determine cause of death. Cause of death was categorized based on information contained in death certificates as CVD-related (myocardial infarction, congestive heart failure, cardiac arrhythmia, sudden cardiac death, peripheral vascular disease, and stroke) or either cancer, infection, end-stage renal disease, accidental, or other (including obstructive pulmonary disease, pulmonary fibrosis, liver failure and Alzheimer’s dementia).

### Statistical analysis

Summary statistics were determined for key demographic and outcome measures; for dichotomous/ordinal measures these are presented as counts and percentages and for continuous measures as mean ± standard deviation. Continuous variables were transformed as appropriate to approximate conditional normality. In order to compare the relative importance, continuous variables were standardized for analysis of associations with outcome.

Survival curves were constructed to examine patterns of mortality for increasing quintiles of vascular calcified plaque in each of the three vascular beds and using the multi-bed score. Due to the inclusion of related individuals in the DHS, Cox proportional hazards models with sandwich-based variance estimation were used to further examine the relationships between measures of vascular calcified plaque and both all-cause mortality and CVD-mortality. Initially, analyses of the simple univariate associations using vascular calcified plaque quintiles were performed. Each of these associations was subsequently adjusted for (i) age and sex and (ii) the Framingham Risk Score Factors (FRS): age, sex, total cholesterol, HDL-cholesterol, smoking, systolic BP, and anti-hypertensive medication use. Associations were then repeated using vascular calcified plaque scores as continuous variables and again following stratification of the sample for both history of prior CVD and age (<65 years and >65 years).

Receiver operating characteristic curves were computed for models containing Framingham risk factors with addition of either CAC, CarCP, AACP, the multi-bed score or all three vascular beds simultaneously. The areas under the curves were used to assess the ability of measures of vascular calcified plaque to predict all-cause mortality or CVD-mortality, after adjusting for established FRS. The difference in area under the curve between two models was tested using Delong’s method [34]. The net reclassification improvement (NRI) was also determined to measure the degree to which risk for all-cause mortality or CVD mortality was reclassified using the Framingham risk factors with the addition of each of the various vascular calcification scores. The percent of the sample reclassified (either into higher or lower risk groups) was reported.

Analyses were performed using SAS version 9.2 (SAS Institute Inc., Cary, NC) with the exception of the Receiver operating characteristic curve analyses which were performed using Stata software, version 12.1 (StataCorp, College Station, TX). Statistical significance was accepted at p < 0.05.

## Results

### Demographics and general outcomes

This analysis included 699 T2D-affected individuals with complete data for vascular calcified plaque across the three vascular beds and full covariate information. Demographic and clinical characteristics are presented in Table 1. Key demographic characteristics were generally similar between participants included in this analysis and those excluded from the analysis (exclusions due to T2D unaffected status n = 199 and incomplete covariate information n = 313; Additional file 1).

**Table 1:** **Demographic and clinical characteristics of European American Diabetes Heart Study (DHS) participants with complete data on vascular calcified plaque (n = 699)**

	**Mean ± SD or %**	**Median (range)**
**Demographic information**		
Age (years)	62.8 ± 8.6	63.1 (40–86)
Gender (% female)	50.2%	
Diabetes duration (years)	10.3 ± 7.1	8 (0–46)
% smoking (current or past)	59.9%	
Self-reported history of prior CVD (%)	43.8%	
Deceased (%)	22.3%	
Deceased from CVD (%)	10.6%	
**Body composition**		
Height (cm)	168.7 ± 9.6	168.7 (122.8-195.4)
Weight (kg)	92.0 ± 19.6	89.5 (40.8-158.8)
BMI (kg/m^2^)	32.2 ± 6.2	31.3 (17.1-55.6)
**Medications**		
Oral hypoglycemic agents	80.0%	
Lipid lowering (%)	52.9%	
Anti-hypertensive (%)	74.5%	
**Blood pressure (BP)**		
Systolic BP (mmHg)	139 ± 18	138 (94–214)
Diastolic BP (mmHg)	72 ± 10	72 (37–106)
Hypertension (%)	87.8%	
**Blood biochemistry**		
Glucose (mg/dL)	146.3 ± 53.7	135 (16–463)
Hemoglobin A_1C_ (%)	7.4 ± 1.4	7.1 (4.6-16.6)
Hemoglobin A_1C_ (mmol/mol)	57.0 ± 15.7	54.1 (26.8-157.9)
Total Cholesterol (mg/ dL)	183.6 ± 42.0	180 (65–391)
High density lipoprotein cholesterol (mg/dL)	42.7 ± 12.1	41 (8–98)
Low density lipoprotein cholesterol (mg/dL)	102.5 ± 32.2	100 (12–236)
Triglycerides (mg/dL)	198.6 ± 124.3	170 (30–1068)
**Vascular imaging**		
Coronary artery calcified plaque	1893 ± 3515	420 (0–50415)
Carotid artery calcified plaque	346 ± 705	56 (0–5954)
Abdominal aortic calcified plaque	12113 ± 16353	4561 (0–94156)
Multi-bed score	1.95 ± 2.71	0.86 (0–23.57)

As anticipated in a T2D enriched sample, a predominance of conventional CVD risk factors were evident including high BMI, hypertension, dyslipidemia and prior CVD events. Scores for vascular calcified plaque reflect a substantial burden of subclinical CVD. The cohort was followed for 8.4 ± 2.3 years (mean ± standard deviation) during which time 156 (22.3%) participants were deceased, 74 (10.6%) from CVD causes. Based on information contained in available death certificates, CVD causes included coronary artery disease (n = 16), myocardial infarction (n = 21), cardiac arrest (n = 16), congestive heart failure (n = 14) and stroke (n = 7).

### Survival analysis

**Figure 1:**
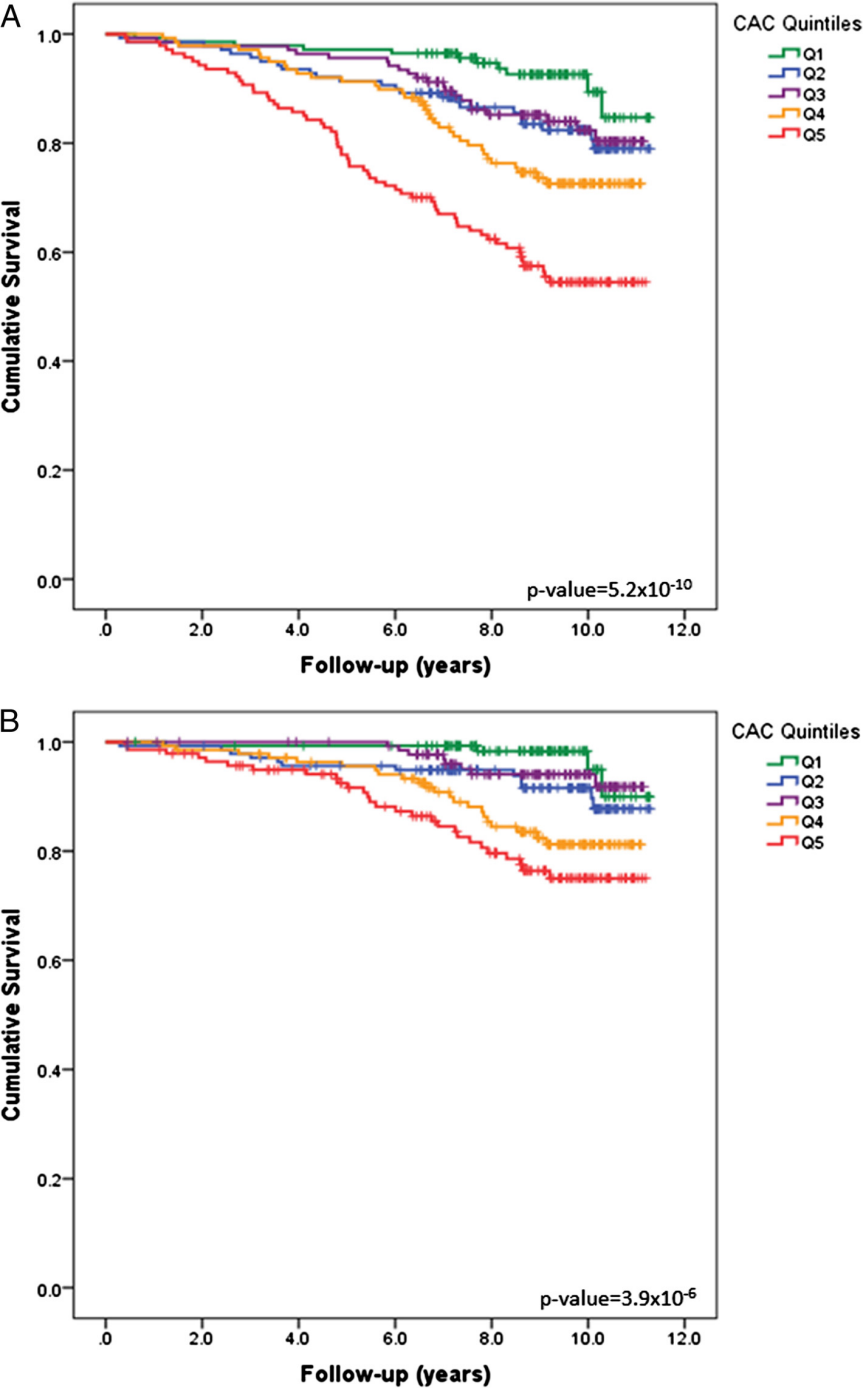
**Survial Curves based on coronary artery calcified plaque burden.** Survival Curves for **(A)** all-cause and **(B)** CVD mortality based on increasing coronary artery calcified plaque (CAC) quintiles.

**Figure 2:**
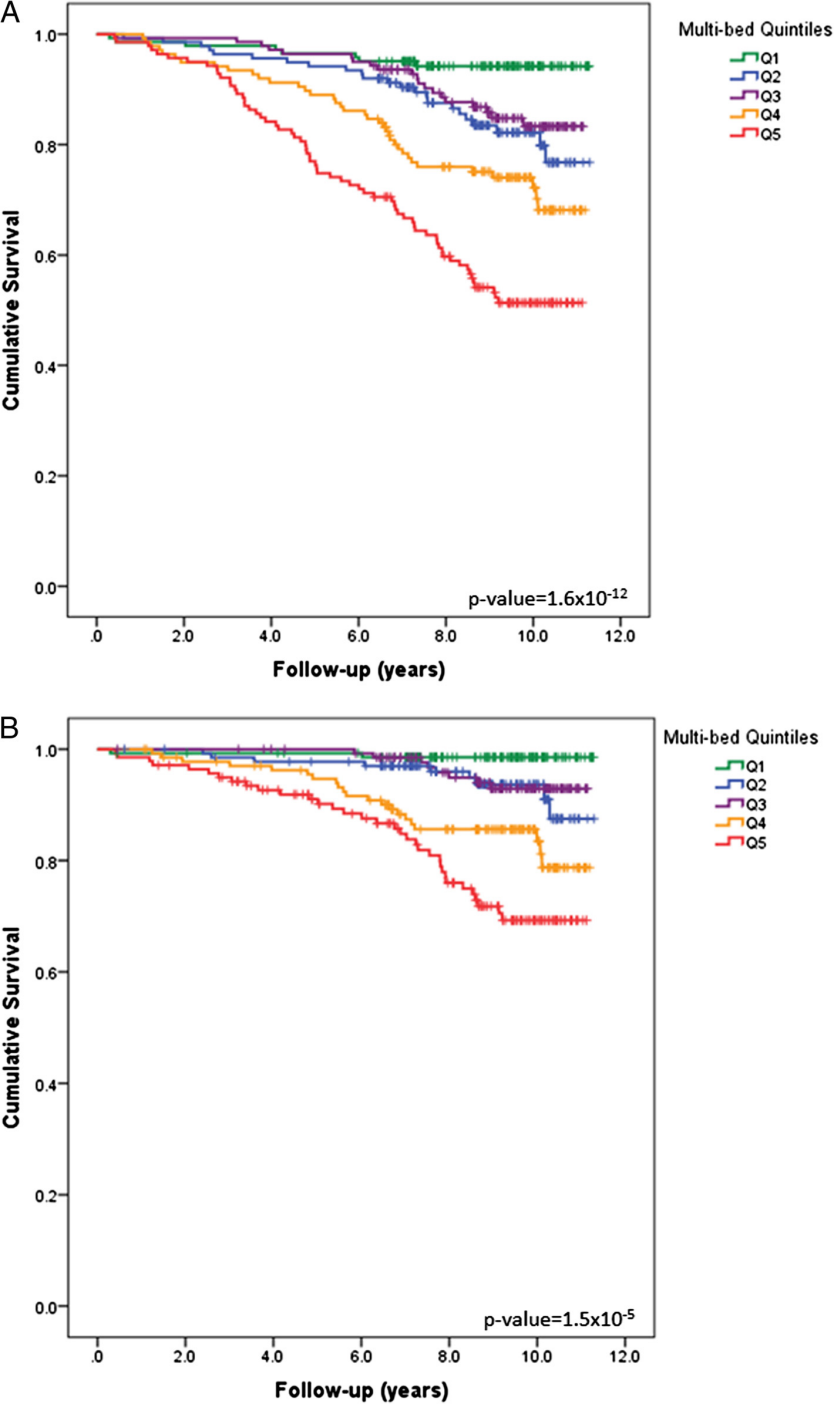
**Survial Curves based on multi-bed calcified plaque burden.** Survival Curves for **(A)** all-cause and **(B)** CVD mortality based on increasing quintiles of the multi-bed vascular calcification score.

A preliminary analysis using survival curves revealed increasing incidence of all-cause and CVD-mortality with increasing vascular calcified plaque quintiles. Compared to the established CAC (Figure 1), these trends initially appeared stronger for the multi-bed score (Figure 2). Increasing risk for all-cause and CVD-mortality was also evident for increasing CarCP and AACP (Additional file 2).

### Cox proportional hazards regression

Further quantification of these effects using Cox proportion hazards regression models confirmed significant associations between all measures of vascular calcified plaque (quintiles) and both all-cause and CVD-mortality (Table 2). These associations remained significant following adjustment for FRS. When considering measures of vascular calcified plaque as continuous variables, simple univariate analysis revealed significant associations between measures of calcification and both all-cause and CVD-mortality (Table 3). For each of the three vascular beds considered individually, each standard deviation unit increase in calcified plaque was associated with a 1.7-2.0 fold (p < 1×10^−9^) increase in risk for all-cause mortality and a 1.9-2.2 fold (p < 1×10^−5^) increase in risk for CVD-mortality. By comparison, the multi-bed score was associated with a 1.8-fold (p < 1×10^−15^) and 2.0-fold (p < 1×10^−10^) increase in risk for all-cause and CVD-mortality respectively; that is improved precision of the estimate, but not outperforming CAC which showed the strongest association.

**Table 2:** **Association between vascular calcified plaque quintiles and mortality in DHS participants based on Cox Proportional Hazards regression**

	**Model 1**		**Model 2**		**Model 3**	
**All-cause mortality**						
	**HR (95% CI)**	**p-value**	**HR (95% CI)**	**p-value**	**HR (95% CI)**	**p-value**
**CAC**	1.57 (1.34-1.76)	5.22 × 10^−10^	1.45 (1.24-1.69)	2.42 × 10^−6^	1.39 (1.18-1.63)	6.55 × 10^−5^
**CarCP**	1.58 (1.40-1.78)	1.57 × 10^−13^	1.41 (1.24-1.60)	1.23 × 10^−7^	1.36 (1.20-1.55)	3.28 × 10^−6^
**AACP**	1.55 (1.36-1.77)	6.22 × 10^−11^	1.37 (1.20-1.56)	3.80 × 10^−6^	1.30 (1.14-1.49)	0.0001
**Multi-bed**	1.65 (1.44-1.90)	1.60 × 10^−12^	1.49 (1.28-1.72)	1.70 × 10^−7^	1.42 (1.22-1.66)	6.11 × 10^−6^
**CVD mortality**						
**CAC**	1.60 (1.31-1.96)	3.86 × 10^−6^	1.47 (1.18-1.83)	0.0007	1.40 (1.12-1.75)	0.003
**CarCP**	1.72 (1.44-2.07)	5.92 × 10^−9^	1.49 (1.23-1.81)	4.83 × 10^−5^	1.46 (1.20-1.77)	0.0001
**AACP**	1.73 (1.39-2.16)	7.77 × 10^−7^	1.49 (1.18-1.88)	0.0009	1.44 (1.14-1.81)	0.002
**Multi-bed**	1.91 (1.52-2.39)	1.87 × 10^−8^	1.68 (1.33-2.12)	1.57 × 10^−5^	1.63 (1.29-2.07)	5.11 × 10^−5^

Hazard Ratios (HR) and confidence intervals (CI) are for each incremental increase in calcified plaque quintiles.

Model 1: unadjusted; Model 2: adjusted for age and sex; Model 3: adjusted for age, sex, total cholesterol, HDL-cholesterol, smoking, systolic blood pressure, and anti-hypertensive medication use.

CAC: coronary artery calcified plaque; CarCP: carotid artery calcified plaque; AACP – abdominal aortic calcified plaque.

**Table 3:** **Association between vascular calcified plaque scores and mortality in DHS participants based on Cox Proportional Hazards regression**

	**Model 1**		**Model 2**		**Model 3**	
**All-cause mortality**						
	**HR (95% CI)**	**p-value**	**HR (95% CI)**	**p-value**	**HR (95% CI)**	**p-value**
**CAC**	2.02 (1.63-2.52)	2.58 × 10^−10^	1.87 (1.45-2.41)	1.31 × 10^−6^	1.76 (1.36-2.27)	2.01 × 10^−5^
**CarCP**	1.93 (1.61-2.32)	2.42 × 10^−12^	1.62 (1.34-1.96)	5.81 × 10^−7^	1.54 (1.27-1.87)	1.37 × 10^−5^
**AACP**	1.72 (1.51-1.97)	3.55 × 10^−15^	1.45 (1.24-1.69)	2.24 × 10^−6^	1.39 (1.18-1.62)	5.40 × 10^−5^
**Multi-bed**	1.81 (1.57-2.09)	2.22 × 10^−16^	1.60 (1.36-1.88)	1.72 × 10^−8^	1.52 (1.29-1.81)	1.13 × 10^−6^
**CVD mortality**						
**CAC**	2.24 (1.60-3.13)	2.24 × 10^−6^	2.01 (1.38-2.93)	0.0003	1.88 (1.29-2.73)	0.001
**CarCP**	2.20 (1.65-2.94)	6.62 × 10^−8^	1.76 (1.31-2.36)	0.0002	1.71 (1.27-2.29)	0.0004
**AACP**	1.91 (1.57-2.32)	1.01 × 10^−10^	1.53 (1.20-1.95)	0.0006	1.51 (1.19-1.92)	0.0009
**Multi-bed**	2.00 (1.63-2.46)	2.73 × 10^−11^	1.71 (1.34-2.18)	1.26 × 10^−5^	1.67 (1.32-2.13)	2.62 × 10^−5^

Calcified plaque scores are considered as continuous variables and standardized to compare relative effects.

Model 1: unadjusted; Model 2: adjusted for age and sex; Model 3: adjusted for age, sex, total cholesterol, HDL-cholesterol, smoking, systolic blood pressure, and anti-hypertensive medication use.

CAC: coronary artery calcified plaque; CarCP: carotid artery calcified plaque; AACP – abdominal aortic calcified plaque.

Associations with outcome remained significant after adjustment for FRS, although magnitudes were decreased; 1.4-1.8 fold (p < 1×10^−5^) elevations in risk for all-cause and 1.5-1.9-fold (p < 1×10^−4^) elevations in risk for CVD-mortality. Similar patterns were again observed for the multi-bed score, with improved precision relative to the single beds (Table 3), although the strength of the associations were not greater than those observed for CAC.

Stratification of the sample by history of prior CVD and age revealed differences in the prevalence of both all-cause mortality (no prior CVD: 14.8%, prior CVD: 31.5%; <65 years: 14.3%, >65 years: 34.5%) and CVD mortality (no prior CVD: 5.4%, prior CVD: 16.9%; <65 years: 5.5%, >65 years: 18.3%). Vascular calcification scores remained significantly associated with outcome following stratification by both history of prior CVD and age, even following adjustment for FRS factors (Additional file 3). Interestingly, stratification of the sample by history of prior CVD revealed stronger associations (in fully adjusted models) for individuals without history of prior CVD (multi-bed score all-cause mortality HR: 1.78; CVD-mortality HR: 2.28) compared to those with a history of prior CVD (all-cause mortality HR: 1.31; CVD-mortality HR: 1.33). These patterns were not as obvious when stratifying by age.

### Area under the curve and net reclassification improvement

Area under the curve (AUC) analysis revealed that regardless of the vascular bed, addition of vascular calcified plaque scores to models including FRS significantly improved prediction of outcome (Table 4). Considering the single beds in isolation, the best gains were achieved upon addition of CAC; for all-cause mortality the AUC increased from 0.720 to 0.757 (p = 0.004) and for CVD-mortality the AUC increased from 0.731 to 0.763 (p = 0.04) upon addition of CAC. For all-cause mortality, the multi-bed score did not outperform CAC (AUC: 0.757), although simultaneous addition of all vascular beds did improve prediction increasing the AUC to 0.764 (p = 0.002). In contrast, for CVD-mortality, the model including the multi-bed score did nominally outperformed CAC (AUC: 0.762 to 0.767; p = 0.008) with significant improvements also noted with simultaneous addition of all vascular beds (AUC: 0.763 to 0.771; p = 0.01).

**Table 4:** **Area under the curve (AUC) analysis comparing the utility of measures of vascular calcification in the prediction of all-cause and CVD mortality**

**Model**	**Covariates**	**AUC**	**Comparison**	**p-value**
**All-cause Mortality**				
1	FRS	0.7203	-	-
2	FRS + CAC	0.7571	1 vs. 2	0.0044
3	FRS + CarCP	0.7493	1 vs. 3	0.0082
4	FRS + AACP	0.7447	1 vs. 4	0.0068
5	FRS + Multi-bed	0.7545	1 vs. 5	0.0031
6	FRS + CAC + CarCP + AACP	0.7644	1 vs. 6	0.0015
**CVD Mortality**				
1	FRS	0.7314	-	-
2	FRS + CAC	0.7625	1 vs. 2	0.0427
3	FRS + CarCP	0.7578	1 vs. 3	0.0459
4	FRS + AACP	0.7582	1 vs. 4	0.0270
5	FRS + Multi-bed	0.7674	1 vs. 5	0.0083
6	FRS + CAC + CarCP + AACP	0.7709	1 vs. 6	0.0133

Models were established with Framingham risk score (FRS) factors (age, sex, total cholesterol, HDL-cholesterol, smoking, systolic blood pressure, anti-hypertensive medication use) and addition of calcified plaque scores.

CAC: coronary artery calcified plaque; CarCP: carotid artery calcified plaque; AACP: abdominal aortic calcified plaque.

The NRI index also suggested the addition of the vascular calcification scores substantially improved risk prediction. Broadly speaking, the NRI indicated that up to 30% of the sample was reclassified depending on the model (Additional file 4). Addition of each of the vascular beds to the Framingham risk factors significantly improved prediction of both all-cause mortality (NRI: 0.100-0.149; p = 0.002-0.024) and CVD mortality (NRI: 0.117-0.149; p = 0.005-0.047).

### High-risk individuals

Given the utility of vascular calcified plaque in predicting outcome, risk for mortality was further quantified for those individuals with the greatest burden of subclinical CVD (Additional file 5). Following adjustment for FRS, individuals with calcified plaque scores in the upper quintile of the CAC distribution experienced a 4.3-fold (p = 0.0003) increase risk of all-cause mortality when compared to those in the lowest quintile; for CarCP, a 4.5-fold (p = 3.0×10^−5^) increase in risk; for AACP, a 2.3-fold (p = 0.01) increase in risk; and for the multi-bed score, a 4.6-fold increase in risk (p = 6.2×10^−5^). For CVD-mortality, individuals with calcified plaque scores in the upper quintile of the CAC distribution experienced a 4.6-fold (p = 0.01) increase in risk compared to those in the lowest quintile; for CarCP, an 8.0-fold (p = 0.005) increase in risk; for AACP, a 3.8-fold (p = 0.04) increase in risk; and for the multi-bed score, a 9.7-fold increase in risk (p = 0.002).

## Discussion

This study, focused on T2D-affected European Americans from the DHS, examined prediction of all-cause and CVD-mortality using measures of vascular calcified plaque. We have previously demonstrated that CAC powerfully predicted outcome in this sample [6,19] and we now extend these observations by examining calcified plaque from additional vascular beds. In addition to CAC, both CarCP and AACP, were independent predictors of mortality and significantly improved prediction beyond the FRS. Finally, use of a derived score to reflect global vascular calcified plaque allowed for improved precision when predicting outcome although it did not outperform the established predictor CAC.

Given the attention CAC has received in the existing literature, it was not surprising that in this sample of T2D-affected individuals, associations with all-cause mortality and CVD-mortality were strongest for CAC. For all-cause mortality, each standard deviation increment increase in CAC was associated with a ~1.8-fold increase in risk, compared to ~1.5-fold and ~1.4-fold increases for CarCP and AACP respectively. The same trend was also observed for risk for CVD-mortality (CAC ~1.9-fold increase in risk; CarCP ~1.7-fold; AACP ~1.5-fold). These findings differ from those recently reported by Allison et al., where AACP was found to be a stronger predictor of both all-cause (hazard ratio (HR) per standard deviation unit increment in calcification: 1.50) and CVD-mortality (HR: 1.62) compared to CAC (HR: 1.22 and 1.33) and CarCP (HR: 1.35 and 1.23) [25]. These differing outcomes may be accounted for in part by the low prevalence of T2D (3%), the low prevalence of vascular calcification (32-56%) and low mortality rate (~3% all-cause, <1% CVD) in the earlier study by Allison et al. compared to the DHS cohort (100% T2D affected; 78-96% with vascular calcification; 22% mortality). Further comparison with the literature is difficult. We are aware of few other studies that compare calcified plaque burden from coronary, carotid and aortic vascular beds directly and those studies that have measured calcification in multiple vascular beds frequently used alternate sites, namely the thoracic aorta [35], have utilized methodologies other the CT scan [36], or focused on association with outcomes other than mortality [37].

An additional element of this study was use of a derived multi-bed calcification score. We previously reported the partial correlation between vascular calcified plaque in the coronary, carotid and abdominal aortic vascular beds (Spearman correlation coefficients 0.6-0.7) [31] supporting the possibility that consideration of all three vascular beds in conjunction may capture more of the phenotypic variance and thus allow for improved risk prediction. While the multi-bed score was not associated with a greater risk for either all-cause or CVD-mortality compared to CAC in analyses using calcified plaque as a continuous trait, the significance of the associations were greater for the multi-bed score suggesting an improved precision of the estimate. Of further interest, AUC analysis revealed that consideration of vascular calcified plaque more globally (either as a multi-bed score or simultaneous inclusion of the three vascular beds) does offer the opportunity for improved prediction of outcome. Here this was most evident for CVD-mortality. Given the different causes of CVD death in this cohort, the multiple vascular beds may better capture risk for both true coronary events and other outcomes, including fatal stroke. Indeed utility of CarCP in predicting risk for stroke has been previously reported [38]. Thus, a more global assessment of the burden of calcified plaque may improve prediction of CVD-mortality beyond any single vascular bed.

Finally, when quantifying risk for adverse outcome in individuals with calcified plaque scores in the upper quintile for each of the vascular beds, those in the upper quintile of the multi-bed score fared worse for both all-cause and CVD-mortality. This is not surprising given that the multi-bed score should better account for individuals with a burden of vascular calcified plaque across multiple beds. From this perspective the multi-bed score should more fully reflect the extent of subclinical disease and be of use in identifying individuals at greatest risk for adverse outcome. That said, the present findings do not support wide-spread need for additional imaging procedures to capture all three vascular beds; CAC alone is likely adequate for general risk stratification purposes. However, if information from multiple vascular beds can be ascertained from existing diagnostic imaging, then additional consideration of the vascular calcified plaque more globally may be warranted. Indeed the analysis stratified by history of prior CVD, suggested that additional risk stratification could be most informative in individuals who might otherwise be overlooked when considering more traditional CVD risk factors.

## Conclusions

This study confirms the utility of vascular calcified plaque as an independent predictor of both all-cause and CVD-mortality in T2D-affected individuals. Although CAC was the strongest predictor of outcome in this cohort, CarCP and AACP also significantly improved prediction of outcome beyond traditional risk factors and should not be discounted as risk stratification tools. Consideration of vascular calcified plaque more globally using a derived multi-bed score may improve precision by accounting for more of the phenotypic variance, however, additional imaging procedures for the purpose of general risk stratification do not appear warranted.

## Additional files

Additional file 1:
**Demographic characteristics for included and excluded participants.**
Additional file 2:
**Survival curves for coronary artery calcified plaque and abdominal aortic calcified plaque.**
Additional file 3:
**Prediction of outcome stratified by history of prior CVD and age.**
Additional file 4:
**Net reclassification index for vascular calcified plaque scores.**
Additional file 5:
**Prediction of outcome for increasing vascular calcified plaque quintiles.**

## Abbreviations

AACP: Abdominal aortic calcified plaque
BP: Blood pressure
CAC: Coronary artery calcified plaque
CarCP: Carotid artery calcified plaque
CT: Computed tomography
CVD: Cardiovascular disease
DHS: Diabetes Heart Study
FRS: Framingham risk score factors
HR: Hazard ratio
T2D: Type 2 diabetes

## References

[1] Roger VL, Go AS, Lloyd-Jones DM, Benjamin EJ, Berry JD, Borden WB, Bravata DM, Dai S, Ford ES, Fox CS, Fullerton HJ, Gillespie C, Hailpern SM, Heit JA, Howard VJ, Kissela BM, Kittner SJ, Lackland DT, Lichtman JH, Lisabeth LD, Makuc DM, Marcus GM, Marelli A, Matchar DB, Moy CS, Mozaffarian D, Mussolino ME, Nichol G, Paynter NP, Soliman EZ (2012). Heart disease and stroke statistics–2012 update: a report from the American Heart Association. Circulation.

[2] Centers for Disease Control and Prevention: National Diabetes Fact Sheet, 2011. http://www.cdc.gov/diabetes/pubs/pdf/ndfs_2011.pdf

[3] Paynter NP, Mazer NA, Pradhan AD, Gaziano JM, Ridker PM, Cook NR (2011). Cardiovascular risk prediction in diabetic men and women using hemoglobin A1c vs diabetes as a high-risk equivalent. Arch Intern Med.

[4] Silverman MG, Blaha MJ, Budoff MJ, Rivera JJ, Raggi P, Shaw LJ, Berman D, Callister T, Rumberger JA, Rana JS, Blumenthal RS, Nasir K (2012). Potential implications of coronary artery calcium testing for guiding aspirin use among asymptomatic individuals with diabetes. Diabetes Care.

[5] Bartels DW, Davidson MH, Gong WC (2007). Type 2 diabetes and cardiovascular disease: reducing the risk. JMCP.

[6] Agarwal S, Morgan T, Herrington DM, Xu J, Cox AJ, Freedman BI, Carr JJ, Bowden DW (2011). Coronary calcium score and prediction of all-cause mortality in diabetes: the Diabetes Heart Study. Diabetes Care.

[7] Hoff JA, Quinn L, Sevrukov A, Lipton RB, Daviglus M, Garside DB, Ajmere NK, Gandhi S, Kondos GT (2003). The prevalence of coronary artery calcium among diabetic individuals without known coronary artery disease. J Am Coll Cardiol.

[8] Arad Y, Newstein D, Cadet F, Roth M, Guerci AD (2001). Association of multiple risk factors and insulin resistance with increased prevalence of asymptomatic coronary artery disease by an electron-beam computed tomographic study. Arterioscler Thromb Vasc Biol.

[9] Brown ER, Kronmal RA, Bluemke DA, Guerci AD, Carr JJ, Goldin J, Detrano R (2008). Coronary calcium coverage score: determination, correlates, and predictive accuracy in the Multi-Ethnic Study of Atherosclerosis. Radiology.

[10] Detrano R, Guerci AD, Carr JJ, Bild DE, Burke G, Folsom AR, Liu K, Shea S, Szklo M, Bluemke DA, O'Leary DH, Tracy R, Watson K, Wong ND, Kronmal RA (2008). Coronary calcium as a predictor of coronary events in four racial or ethnic groups. N Engl J Med.

[11] Folsom AR, Kronmal RA, Detrano RC, O'Leary DH, Bild DE, Bluemke DA, Budoff MJ, Liu K, Shea S, Szklo M, Tracy RP, Watson KE, Burke GL (2008). Coronary artery calcification compared with carotid intima-media thickness in the prediction of cardiovascular disease incidence: the Multi-Ethnic Study of Atherosclerosis (MESA). Arch Intern Med.

[12] Shemesh J, Tenenbaum A, Fisman EZ, Koren-Morag N, Grossman E (2013). Coronary calcium in patients with and without diabetes: first manifestation of acute or chronic coronary events is characterized by different calcification patterns. Cardiovasc Diabetol.

[13] Lau KK, Wong YK, Chan YH, Yiu KH, Teo KC, Li LS, Ho SL, Chan KH, Siu CW, Tse HF (2012). Prognostic implications of surrogate markers of atherosclerosis in low to intermediate risk patients with type 2 diabetes. Cardiovasc Diabetol.

[14] Raggi P, Shaw LJ, Berman DS, Callister TQ (2004). Prognostic value of coronary artery calcium screening in subjects with and without diabetes. J Am Coll Cardiol.

[15] Kramer CK, Zinman B, Gross JL, Canani LH, Rodrigues TC, Azevedo MJ, Retnakaran R (2013). Coronary artery calcium score prediction of all cause mortality and cardiovascular events in people with type 2 diabetes: systematic review and meta-analysis. BMJ.

[16] Elkeles RS, Godsland IF, Feher MD, Rubens MB, Roughton M, Nugara F, Humphries SE, Richmond W, Flather MD (2008). Coronary calcium measurement improves prediction of cardiovascular events in asymptomatic patients with type 2 diabetes: the PREDICT study. Eur Heart J.

[17] Chiu YW, Adler SG, Budoff MJ, Takasu J, Ashai J, Mehrotra R (2010). Coronary artery calcification and mortality in diabetic patients with proteinuria. Kidney Int.

[18] Anand DV, Lim E, Hopkins D, Corder R, Shaw LJ, Sharp P, Lipkin D, Lahiri A (2006). Risk stratification in uncomplicated type 2 diabetes: prospective evaluation of the combined use of coronary artery calcium imaging and selective myocardial perfusion scintigraphy. Eur Heart J.

[19] Agarwal S, Cox AJ, Herrington DM, Jorgensen NW, Xu J, Freedman BI, Carr JJ, Bowden DW (2013). Coronary Calcium Score Predicts Cardiovascular Mortality in Diabetes: Diabetes Heart Study. Diabetes Care.

[20] Isgum I, Rutten A, Prokop M, Staring M, Klein S, Pluim JP, Viergever MA, van Ginneken B (2010). Automated aortic calcium scoring on low-dose chest computed tomography. Med Phys.

[21] Chuang ML, Gona P, Oyama-Manabe N, Manders ES, Salton CJ, Hoffmann U, Manning WJ, O'Donnell CJ (2014). Risk factor differences in calcified and noncalcified aortic plaque: the Framingham Heart Study. Arterioscler Thromb Vasc Biol.

[22] Das M, Braunschweig T, Muhlenbruch G, Mahnken AH, Krings T, Langer S, Koeppel T, Jacobs M, Gunther RW, Mommertz G (2009). Carotid plaque analysis: comparison of dual-source computed tomography (CT) findings and histopathological correlation. Eur J Vasc Endovasc Surg.

[23] Kim ED, Kim JS, Kim SS, Jung JG, Yun SJ, Kim JY, Ryu JS (2013). Association of abdominal aortic calcification with lifestyle and risk factors of cardiovascular disease. Korean J Family Med.

[24] Fanning NF, Walters TD, Fox AJ, Symons SP (2006). Association between calcification of the cervical carotid artery bifurcation and white matter ischemia. AJNR Am J Neuroradiol.

[25] Allison MA, Hsi S, Wassel CL, Morgan C, Ix JH, Wright CM, Criqui MH (2012). Calcified atherosclerosis in different vascular beds and the risk of mortality. Arterioscler Thromb Vasc Biol.

[26] Bowden DW, Lehtinen AB, Ziegler JT, Rudock ME, Xu J, Wagenknecht LE, Herrington DM, Rich SS, Freedman BI, Carr JJ, Langefeld CD (2008). Genetic epidemiology of subclinical cardiovascular disease in the diabetes heart study. Ann Hum Genet.

[27] Bowden DW, Cox AJ, Freedman BI, Hugenschimdt CE, Wagenknecht LE, Herrington D, Agarwal S, Register TD, Maldjian JA, Ng MC, Hsu FC, Langefeld CD, Williamson JD, Carr JJ (2010). Review of the Diabetes Heart Study (DHS) family of studies: a comprehensively examined sample for genetic and epidemiological studies of type 2 diabetes and its complications. Rev Diabet Stud.

[28] Expert Panel on Detection E, and Treatment of High Blood Cholesterol in Adults (2001). Executive Summary of the Third Report of the National Cholesterol Education Program (NCEP) Expert Panel on Detection, Evaluation, and Treatment of High Blood Cholesterol in Adults (Adult Treatment Panel III). JAMA.

[29] Carr JJ, Crouse JR, Goff DC, D'Agostino RB, Peterson NP, Burke GL (2000). Evaluation of subsecond gated helical CT for quantification of coronary artery calcium and comparison with electron beam CT. AJR Am J Roentgenol.

[30] Carr JJ, Nelson JC, Wong ND, McNitt-Gray M, Arad Y, Jacobs DR, Sidney S, Bild DE, Williams OD, Detrano RC (2005). Calcified coronary artery plaque measurement with cardiac CT in population-based studies: standardized protocol of Multi-Ethnic Study of Atherosclerosis (MESA) and Coronary Artery Risk Development in Young Adults (CARDIA) study. Radiology.

[31] Wagenknecht LE, Langefeld CD, Freedman BI, Carr JJ, Bowden DW (2007). A comparison of risk factors for calcified atherosclerotic plaque in the coronary, carotid, and abdominal aortic arteries: the Diabetes Heart Study. Am J Epidemiol.

[32] Carr JJ, Register TC, Hsu FC, Lohman K, Lenchik L, Bowden DW, Langefeld CD, Xu J, Rich SS, Wagenknecht LE, Freedman BI (2008). Calcified atherosclerotic plaque and bone mineral density in type 2 diabetes: the Diabetes Heart Study. Bone.

[33] Ding J, Kritchevsky SB, Hsu FC, Harris TB, Burke GL, Detrano RC, Szklo M, Criqui MH, Allison M, Ouyang P, Brown ER, Carr JJ (2008). Association between non-subcutaneous adiposity and calcified coronary plaque: a substudy of the Multi-Ethnic Study of Atherosclerosis. Am J Clin Nutr.

[34] DeLong ER, DeLong DM, Clarke-Pearson DL (1988). Comparing the areas under two or more correlated receiver operating characteristic curves: a nonparametric approach. Biometrics.

[35] Kalsch H, Lehmann N, Berg MH, Mahabadi AA, Mergen P, Mohlenkamp S, Bauer M, Kara K, Dragano N, Hoffmann B, Moebus S, Schmermund A, Stang A, Jockel KH, Erbel R (2014). Coronary artery calcification outperforms thoracic aortic calcification for the prediction of myocardial infarction and all-cause mortality: The Heinz Nixdorf Recall Study. Eur J Prevent Cardiol.

[36] Wilson PW, Kauppila LI, O'Donnell CJ, Kiel DP, Hannan M, Polak JM, Cupples LA (2001). Abdominal aortic calcific deposits are an important predictor of vascular morbidity and mortality. Circulation.

[37] Elias-Smale SE, Wieberdink RG, Odink AE, Hofman A, Hunink MG, Koudstaal PJ, Krestin GP, Breteler MM, van der Lugt A, Witteman JC (2011). Burden of atherosclerosis improves the prediction of coronary heart disease but not cerebrovascular events: the Rotterdam Study. Eur Heart J.

[38] Elias-Smale SE, Odink AE, Wieberdink RG, Hofman A, Hunink MG, Krestin GP, Koudstaal PJ, Breteler MM, van der Lugt A, Witteman JC (2010). Carotid, aortic arch and coronary calcification are related to history of stroke: the Rotterdam Study. Atherosclerosis.

