# Supplementary material for: Prediction of mortality using a multi-bed vascular calcification score in the Diabetes Heart Study
Source: Cardiovasc Diabetol. 2014 Dec 12;13:160. doi: 10.1186/s12933-014-0160-5 (PMC4266952; doi:10.1186/s12933-014-0160-5)
Supplement: Additional file 1: — Demographic characteristics for included and excluded participants. [file 12933_2014_160_MOESM1_ESM.pdf]

Additional File 1

Demographic characteristics of European American Diabetes Heart Study (DHS) participants included in the analysis and those excluded from the analysis. Exclusions due to T2D unaffected status n=199 and incomplete covariate information n=313.

	Mean ± SD or %	
	Included (n=699)	Excluded (n=521)
**Demographic Information**		
Age (years)	62.8 ± 8.6	61.0 ± 10.2
Gender (% female)	50.2%	58.2%
Diabetes duration (years)	10.3 ± 7.1	10.6 ± 7.3
% smoking (current or past)	59.9%	56.6%
Self-reported history of prior CVD (%)	43.8%	32.6%
Deceased (%)	22.3%	25.5%
Deceased from CVD (%)	10.6%	11.3%
**Body Composition**		
Height (cm)	168.7 ± 9.6	168.3 ± 9.7
Weight (kg)	92.0 ± 19.6	88.5 ± 20.5
BMI (kg/m^2)	32.2 ± 6.2	31.2 ± 6.7
