# Supplementary material for: Prediction of mortality using a multi-bed vascular calcification score in the Diabetes Heart Study
Source: Cardiovasc Diabetol. 2014 Dec 12;13:160. doi: 10.1186/s12933-014-0160-5 (PMC4266952; doi:10.1186/s12933-014-0160-5)

Additional File 2

Survival Curves for (A) all-cause and (B) CVD mortality based on increasing coronary artery calcified plaque (CarCP) quintiles.

(A)

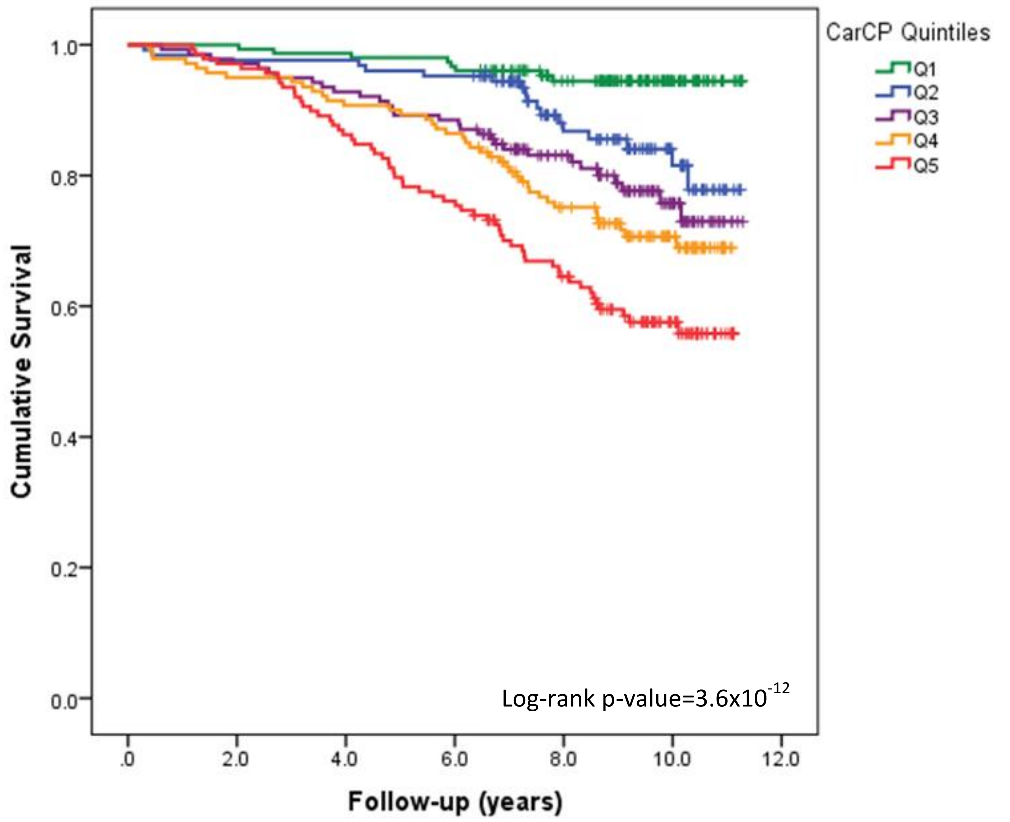

(B)

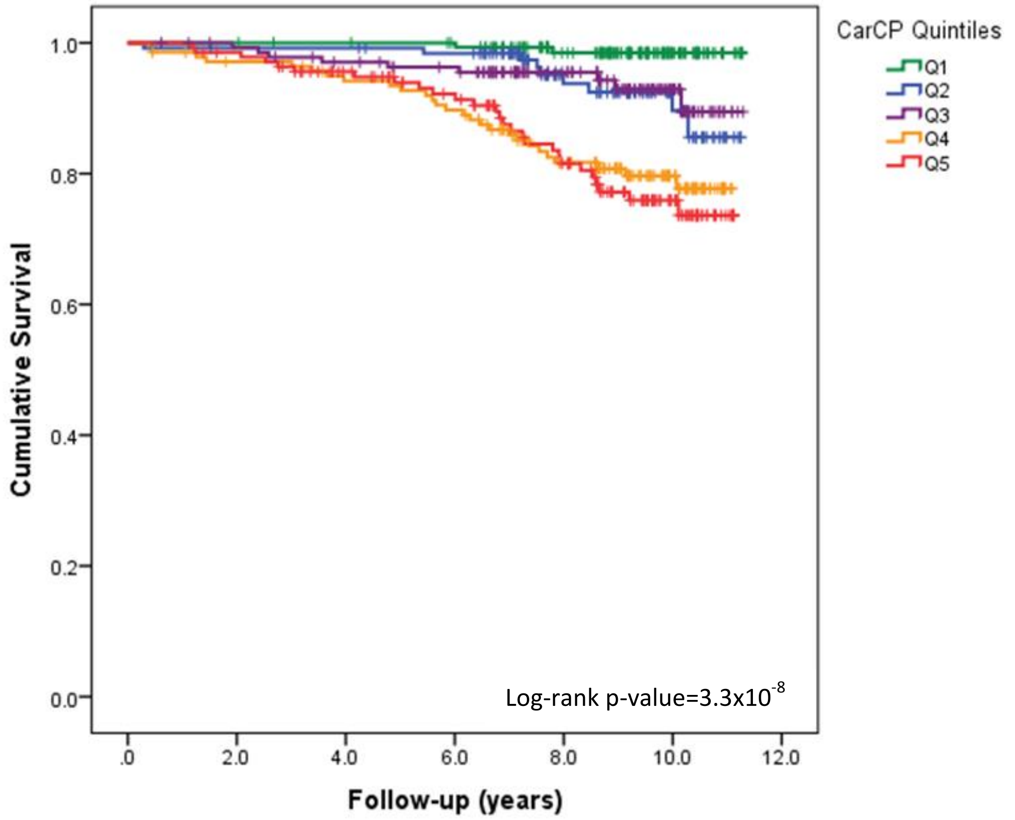

Survival Curves for (A) all-cause and (B) CVD mortality based on increasing abdominal aortic calcified plaque (AACP) quintiles.

(A)

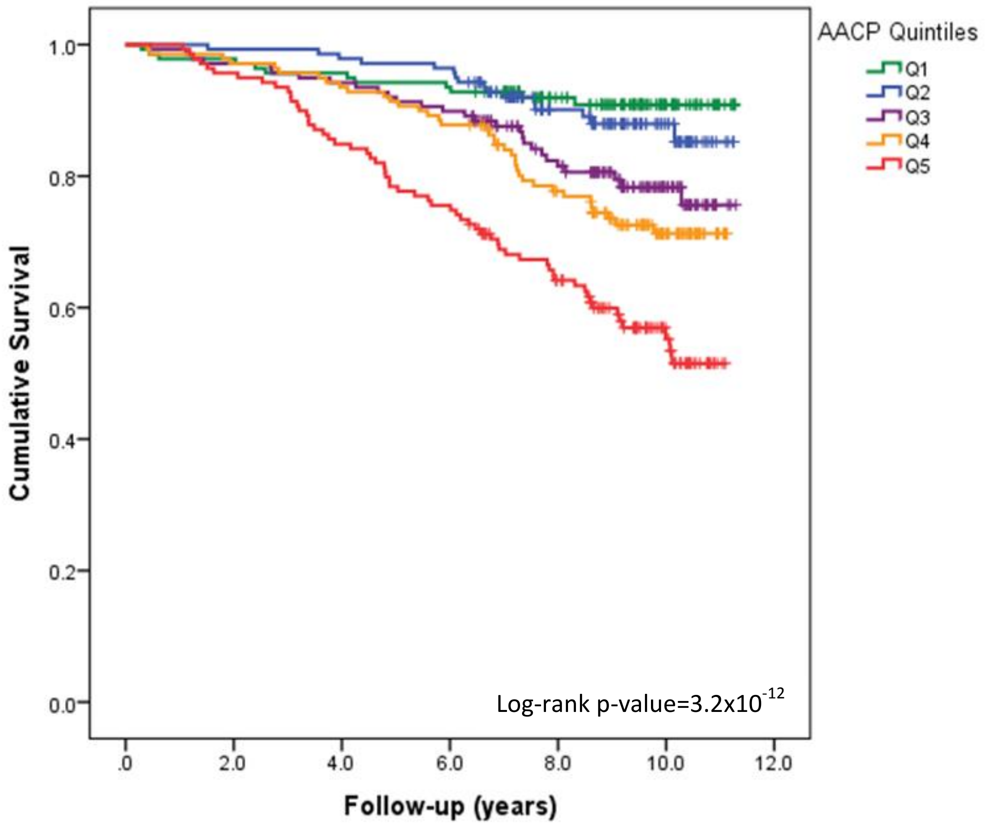

(B)

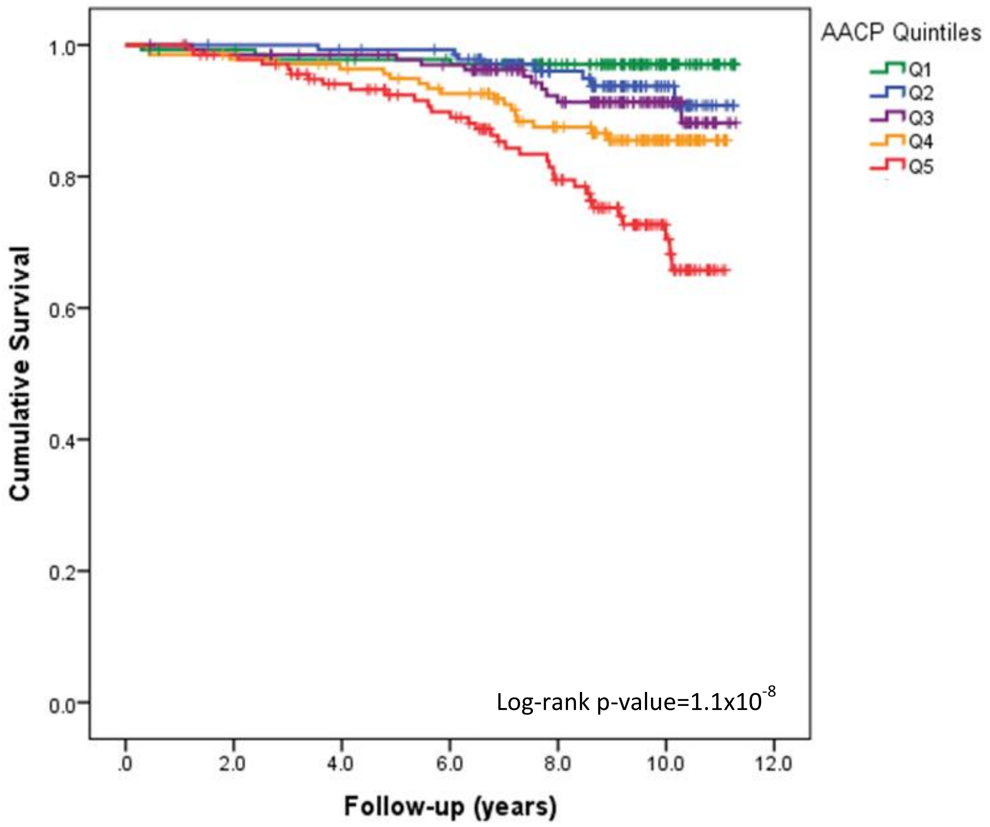

Supplement: Additional file 2: — Survival curves for coronary artery calcified plaque and abdominal aortic calcified plaque. [file 12933_2014_160_MOESM2_ESM.pdf]
