# Supplementary material for: Prediction of mortality using a multi-bed vascular calcification score in the Diabetes Heart Study
Source: Cardiovasc Diabetol. 2014 Dec 12;13:160. doi: 10.1186/s12933-014-0160-5 (PMC4266952; doi:10.1186/s12933-014-0160-5)
Supplement: Additional file 3: — Prediction of outcome stratified by history of prior CVD and age. [file 12933_2014_160_MOESM3_ESM.pdf]

Additional File 3

Association between vascular calcified plaque scores and **all-cause mortality** in DHS participants based on Cox Proportional Hazards regression **stratified by age** (<65 years, >65 years). Calcified plaque scores are considered as continuous variables and standardized to compare relative effects.

	**Model 1**		**Model 2**		**Model 3**	
**Less than 65 years (n=421)**						
	**HR (95% CI)**	**p-value**	**HR (95% CI)**	**p-value**	**HR (95% CI)**	**p-value**
**CAC**	1.57 (1.19-2.09)	0.002	1.67 (1.20-2.32)	0.002	1.51 (1.09-2.10)	0.01
**CarCP**	1.62 (1.23-2.13)	0.0006	1.64 (1.21-2.22)	0.001	1.52 (1.10-2.10)	0.01
**AACP**	1.53 (1.17-2.01)	0.002	1.55 (1.17-2.06)	0.002	1.34 (0.99-1.81)	0.06
**Multi-bed**	1.57 (1.20-2.04)	0.0009	1.63 (1.21-2.19)	0.001	1.46 (1.06-2.01)	0.02
**Greater than 65 years (n=278)**						
**CAC**	2.10 (1.53-2.87)	$3.9x10^{-6}$	2.13 (1.45-3.14)	0.0001	2.01 (1.34-3.00)	0.0007
**CarCP**	1.77 (1.40-2.24)	$2.3x10^{-6}$	1.64 (1.29-2.09)	$5.8x10^{-5}$	1.60 (1.24-2.06)	0.0003
**AACP**	1.52 (1.29-1.80)	$9.6x10^{-7}$	1.38 (1.16-1.66)	0.0004	1.38 (1.14-1.67)	0.0009
**Multi-bed**	1.65 (1.38-1.97)	$2.57x10^{-8}$	1.58 (1.30-1.91)	$3.0x10^{-6}$	1.54 (1.25-1.89)	$4.37x10^{-5}$

Model 1: unadjusted; Model 2: adjusted for age and sex; Model 3: adjusted for age, sex, total cholesterol, HDL-cholesterol, smoking, systolic blood pressure, and anti-hypertensive medication use. CAC: coronary artery calcified plaque; CarCP: carotid artery calcified plaque; AACP – abdominal aortic calcified plaque.

Association between vascular calcified plaque scores and **CVD mortality** in DHS participants based on Cox Proportional Hazards regression **stratified by age** (<65 years, >65 years). Calcified plaque scores are considered as continuous variables and standardized to compare relative effects.

	Model 1		Model 2		Model 3	
**Less than 65 years (n=421)**						
	**HR (95% CI)**	**p-value**	**HR (95% CI)**	**p-value**	**HR (95% CI)**	**p-value**
**CAC**	1.95 (1.12-3.43)	0.02	2.19 (1.19-4.02)	0.01	2.02 (1.13-3.61)	0.02
**CarCP**	1.78 (1.16-2.75)	0.009	1.90 (1.19-3.01)	0.007	1.82 (1.14-2.90)	0.01
**AACP**	2.06 (1.36-3.12)	0.0007	2.25 (1.44-3.51)	0.0004	2.16 (1.40-3.34)	0.0005
**Multi-bed**	1.91 (1.27-2.89)	0.002	2.13 (1.36-3.34)	0.001	2.08 (1.33-3.23)	0.001
**Greater than 65 years (n=278)**						
**CAC**	1.93 (1.32-2.81)	0.0007	1.92 (1.22-3.01)	0.005	1.79 (1.12-2.86)	0.02
**CarCP**	1.86 (1.30-2.64)	0.0006	1.72 (1.19-2.50)	0.004	1.74 (1.17-2.60)	0.007
**AACP**	1.48 (1.16-1.87)	0.001	1.33 (1.03-1.71)	0.03	1.32 (1.00-1.72)	0.05
**Multi-bed**	1.63 (1.28-2.08)	6.4×10^{-5}	1.55 (1.19-2.02)	0.001	1.54 (1.16-2.05)	0.003

Model 1: unadjusted; Model 2: adjusted for age and sex; Model 3: adjusted for age, sex, total cholesterol, HDL-cholesterol, smoking, systolic blood pressure, and anti-hypertensive medication use. CAC: coronary artery calcified plaque; CarCP: carotid artery calcified plaque; AACP – abdominal aortic calcified plaque.

Association between vascular calcified plaque scores and **all-cause mortality** in DHS participants based on Cox Proportional Hazards regression **stratified by history of prior CVD**. Calcified plaque scores are considered as continuous variables and standardized to compare relative effects.

	**Model 1**		**Model 2**		**Model 3**	
**No prior CVD (n=385)**						
	**HR (95% CI)**	**p-value**	**HR (95% CI)**	**p-value**	**HR (95% CI)**	**p-value**
**CAC**	2.26 (1.61-3.18)	2.73×10^{-6}	2.24 (1.46-3.45)	0.0002	2.11 (1.40-3.18)	0.0004
**CarCP**	2.11 (1.58-2.81)	3.7×10^{-7}	1.86 (1.36-2.55)	0.0001	1.75 (1.26-2.43)	0.0009
**AACP**	1.99 (1.54-2.58)	1.5×10^{-7}	1.72 (1.29-2.28)	0.0002	1.61 (1.21-2.14)	0.001
**Multi-bed**	2.02 (1.62-2.52)	5.3×10^{-10}	1.87 (1.43-2.44)	4.3x10-6	1.78 (1.34-2.36)	6.5×10^{-5}
**History of prior CVD (n=314)**						
**CAC**	1.52 (1.13-2.04)	0.006	1.42 (1.04-1.92)	0.03	1.37 (0.98-1.93)	0.07
**CarCP**	1.59 (1.24-2.04)	0.0003	1.35 (1.08-1.70)	0.009	1.33 (1.05-1.68)	0.02
**AACP**	1.45 (1.23-1.71)	1.3×10^{-5}	1.25 (1.06-1.49)	0.01	1.22 (1.02-1.47)	0.03
**Multi-bed**	1.52 (1.25-1.85)	2.8×10^{-5}	1.34 (1.10-1.63)	0.004	1.31 (1.07-1.62)	0.01

Model 1: unadjusted; Model 2: adjusted for age and sex; Model 3: adjusted for age, sex, total cholesterol, HDL-cholesterol, smoking, systolic blood pressure, and anti-hypertensive medication use. CAC: coronary artery calcified plaque; CarCP: carotid artery calcified plaque; AACP – abdominal aortic calcified plaque.

Association between vascular calcified plaque scores and **CVD mortality** in DHS participants based on Cox Proportional Hazards regression **stratified by history of prior CVD**. Calcified plaque scores are considered as continuous variables and standardized to compare relative effects.

	**Model 1**		**Model 2**		**Model 3**	
**No prior CVD (n=385)**						
	**HR (95% CI)**	**p-value**	**HR (95% CI)**	**p-value**	**HR (95% CI)**	**p-value**
**CAC**	2.25 (1.22-4.13)	0.009	1.88 (0.81-4.34)	0.14	1.85 (0.81-4.23)	0.14
**CarCP**	2.82 (1.62-4.93)	0.0003	2.34 (1.31-4.17)	0.004	2.50 (1.30-4.80)	0.006
**AACP**	2.62 (1.61-4.27)	0.0001	2.11 (1.18-3.77)	0.01	2.28 (1.24-4.19)	0.008
**Multi-bed**	2.36 (1.64-3.38)	3.2×10^{-6}	2.05 (1.30-3.25)	0.002	2.28 (1.38-3.75)	0.001
**History of prior CVD (n=314)**						
**CAC**	1.63 (1.10-2.42)	0.02	1.58 (1.09-2.30)	0.02	1.48 (0.99-2.22)	0.06
**CarCP**	1.64 (1.17-2.31)	0.005	1.38 (1.00-1.92)	0.05	1.33 (0.95-1.85)	0.09
**AACP**	1.47 (1.18-1.84)	0.0007	1.26 (0.99-1.60)	0.07	1.20 (0.93-1.54)	0.16
**Multi-bed**	1.58 (1.20-2.06)	0.001	1.39 (1.05-1.83)	0.02	1.33 (1.00-1.76)	0.05

Model 1: unadjusted; Model 2: adjusted for age and sex; Model 3: adjusted for age, sex, total cholesterol, HDL-cholesterol, smoking, systolic blood pressure, and anti-hypertensive medication use. CAC: coronary artery calcified plaque; CarCP: carotid artery calcified plaque; AACP – abdominal aortic calcified plaque.
