# Supplementary material for: Prediction of mortality using a multi-bed vascular calcification score in the Diabetes Heart Study
Source: Cardiovasc Diabetol. 2014 Dec 12;13:160. doi: 10.1186/s12933-014-0160-5 (PMC4266952; doi:10.1186/s12933-014-0160-5)
Supplement: Additional file 4: — Net reclassification index for vascular calcified plaque scores. [file 12933_2014_160_MOESM4_ESM.pdf]

Additional File 4

Changes in risk classification for all-cause mortality and CVD-mortality as assessed using a net reclassification index (NRI) comparing Framingham risk factors and Framingham risk factors with the addition of each of the vascular calcification scores.

Trait	% reclassified	NRI	p-value
**Coronary artery calcified plaque**			
All-cause Mortality	29.3%	0.122	0.009
CVD Mortality	27.5%	0.145	0.005
**Carotid artery calcified plaque**			
All-cause Mortality	30.0%	0.119	0.013
CVD Mortality	30.8%	0.117	0.047
**Abdominal Aortic calcified plaque**			
All-cause Mortality	24.3%	0.100	0.024
CVD Mortality	27.3%	0.149	0.008
**Multi-bed score**			
All-cause Mortality	29.0%	0.149	0.002
CVD Mortality	28.9%	0.149	0.011
**Coronary + Carotid + Abdominal Aortic Calcified Plaque**			
All-cause Mortality	31.9%	0.109	0.027
CVD Mortality	32.0%	0.169	0.004
