# Supplementary material for: Prediction of mortality using a multi-bed vascular calcification score in the Diabetes Heart Study
Source: Cardiovasc Diabetol. 2014 Dec 12;13:160. doi: 10.1186/s12933-014-0160-5 (PMC4266952; doi:10.1186/s12933-014-0160-5)
Supplement: Additional file 5: — Prediction of outcome for increasing vascular calcified plaque quintiles. [file 12933_2014_160_MOESM5_ESM.pdf]

Additional File 5

Association between vascular calcified plaque score quintiles and mortality in DHS participants using models adjusted for age, sex, total cholesterol, HDL-cholesterol, smoking, systolic blood pressure, and use of anti-hypertensive medications. CAC: coronary artery calcified plaque; CarCP: carotid artery calcified plaque; AACP: abdominal aortic calcified plaque.

	CAC			CarCP			AACP			Multi-bed		
**Quintiles**	**Quintile Range**	**HR (95% CI)**	**p-value**	**Quintile Range**	**HR (95% CI)**	**p-value**	**Quintile Range**	**HR (95% CI)**	**p-value**	**Quintile Range**	**HR (95% CI)**	**p-value**
**All-cause mortality**												
**Qu1**	0-29.5	Ref	-	0	Ref	-	0-497	Ref	-	0-0.10	Ref	-
**Qu2**	29.5-214	1.80 (0.84-3.84)	0.13	0-20	2.09 (0.93-4.67)	0.07	497-2713	0.83 (0.38-1.81)	0.64	0.1-0.45	2.07 (0.96-4.46)	0.06
**Qu3**	214-824	1.73 (0.81-3.71)	0.16	20-123	2.71 (1.35-5.45)	0.005	2713-8539	1.38 (0.71-2.66)	0.34	0.45-1.39	1.48 (0.73-2.99)	0.28
**Qu4**	824-3259	2.30 (1.11-4.74)	0.02	123-480	3.19 (1.58-6.46)	0.0012	8539-21858	1.48 (0.76-2.89)	0.25	1.39-3.33	2.85 (1.36-5.98)	0.006
**Qu5**	3295-50415	4.25 (1.94-9.31)	0.0003	480-5954	4.50 (2.22-9.10)	2.96×10^{-5}	21858-94156	2.34 (1.22-4.49)	0.01	3.33-23.57	4.59 (2.18-9.69)	6.24×10^{-5}
**CVD-Mortality**												
**Qu1**	0-29.5	Ref	-	0	Ref	-	0-496.6	Ref	-	0-0.10	Ref	-
**Qu2**	29.5-214	2.28 (0.66-7.94)	0.19	0-20	4.32 (0.89-20.96)	0.07	497-2713	1.28 (0.31-5.30)	0.73	0.1-0.45	3.24 (0.77-13.66)	0.11
**Qu3**	214-824	1.50 (0.44-5.10)	0.51	20-123	3.25 (0.75-14.19)	0.17	2713-8539	1.61 (0.42-0.61)	0.49	0.45-1.39	2.47 (0.53-11.55)	0.25
**Qu4**	824-3259	3.48 (1.14-10.64)	0.03	123-480	8.42 (1.97-35.87)	0.004	8539-21858	2.29 (0.61-8.52)	0.22	1.39-3.33	6.22 (1.45-26.68)	0.01
**Qu5**	3295-50415	4.64 (1.38-15.65)	0.01	480-5954	7.98 (1.89-33.66)	0.005	21858-94156	3.79 (1.05-13.69)	0.04	3.33-23.57	9.67 (2.23-41.97)	0.002
